# A Case of Aortic Valve Infective Endocarditis with Dermatological Findings of Infective Endocarditis

**DOI:** 10.7759/cureus.23044

**Published:** 2022-03-10

**Authors:** Amitoj S Sachdeva, Jorge O Gomez, Keattiyoat Wattanakit

**Affiliations:** 1 Internal Medicine, University of Illinois School of Medicine at Peoria, Peoria, USA; 2 Cardiology, University of Illinois School of Medicine at Peoria, Peoria, USA

**Keywords:** staph aureus endocarditis, osler nodes, janeway lesions, infective endocarditis, aortic valve endocarditis

## Abstract

Infective endocarditis (IE) is associated with high morbidity and mortality. We present a case of a patient that presented with chest pain and had a workup focused on coronary artery disease and acute coronary syndrome. However, the patient had a history and, even more interestingly, physical exam findings, including Janeway lesions, Osler's nodes, and Splinter hemorrhages, indicative of infective endocarditis. We are sharing the findings that raised our suspicion for IE and a discussion on the pathophysiology of these findings in an effort to promote early recognition and treatment of IE.

## Introduction

Although the true incidence of infection endocarditis (IE) is difficult to establish due to variation in the case of definition between authors and clinical centers, Pant et al. [1] found the incidence of IE in the US has increased from 11 to 15 per 100,000 population between 2000 and 2011. The same study by Pant et al. found that the mortality for all cases of definite IE was 20.7%, 26.2%, and 29.2%, respectively, at 30, 90, and 180 days after admission [1]. Therefore, it is important for clinicians to suspect and recognize infective endocarditis to prevent treatment delay and progression of the disease. We present a case of aortic valve endocarditis in a patient that presented with chest pain and had multiple dermatological physical exam findings that raised suspicion of infective endocarditis.

## Case presentation

A 65-year-old male with a past medical history of obstructive sleep apnea, obesity, hypercholesterolemia, hypothyroidism, erectile dysfunction, and hypogonadism presented a few days after a recent visit to the ED with left-sided chest pain that he described as constant 5/10 pressure without radiation and started after a 12 hours long driving trip. The patient underwent CT of the chest that ruled out pulmonary embolism and had serial negative troponin enzyme testing. A stress test was attempted due to risk factors for coronary disease but not completed due to chest pain. Upon further evaluation, the chest pain was not exertional or relieved by rest and treated as chest wall pain with prednisone and nonsteroidal anti-inflammatory drugs (NSAIDs). The patient now reported conversational dyspnea, generalized weakness with fatigue, fevers up to 101℉, chills, and weight loss of 10 lbs over the last few days. Review of systems (ROS) was negative for palpitations, lightheadedness, syncopal episodes, lower extremity swelling, orthopnea, or paroxysmal nocturnal dyspnea (PND). The patient had no recent history of IV drug use and recent surgery or dental procedures.

**Figure 1:**
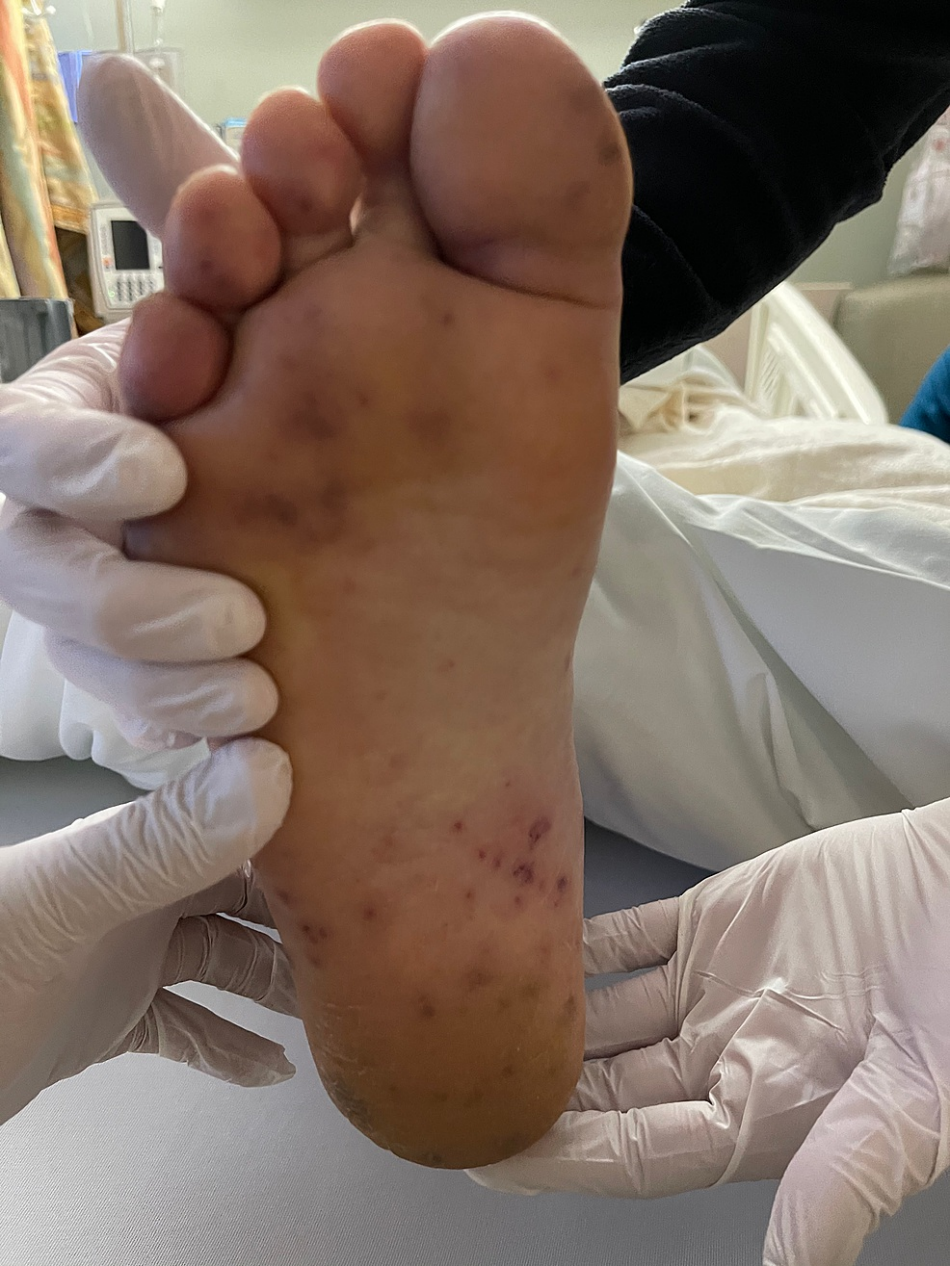
Right foot with multiple non-tender macular erythematous lesions, consistent with Janeway lesions

**Figure 2:**
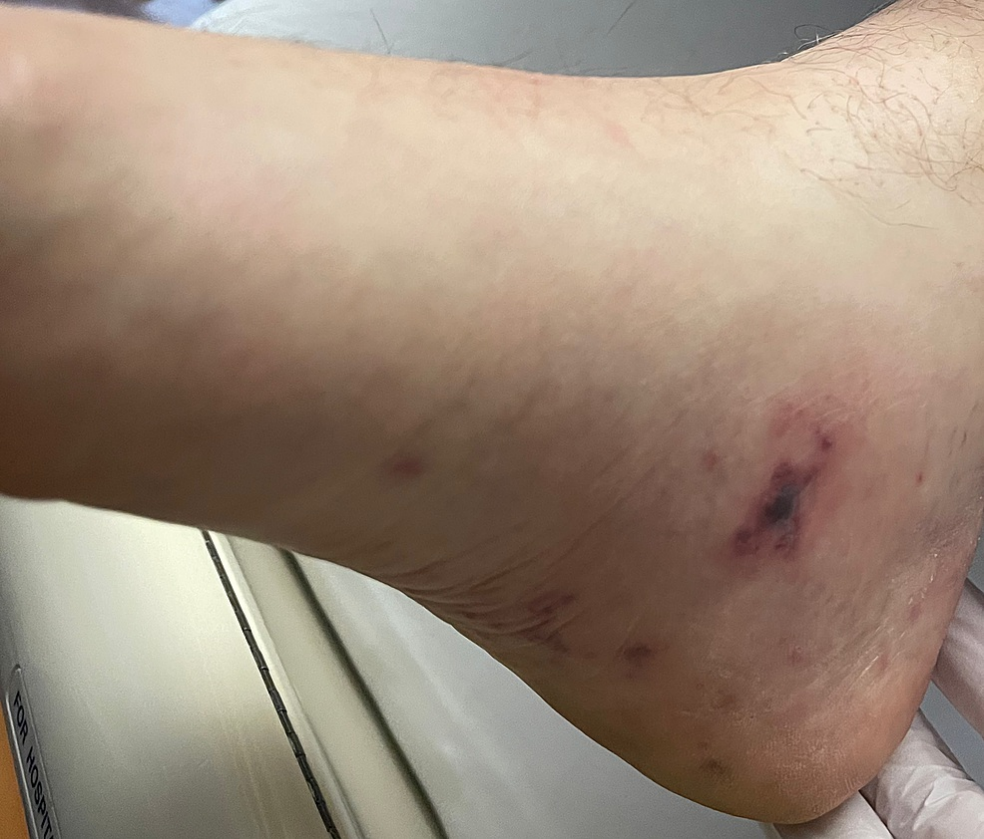
Right foot with a medial tender red-purple lesion, consistent with Osler node

**Figure 3:**
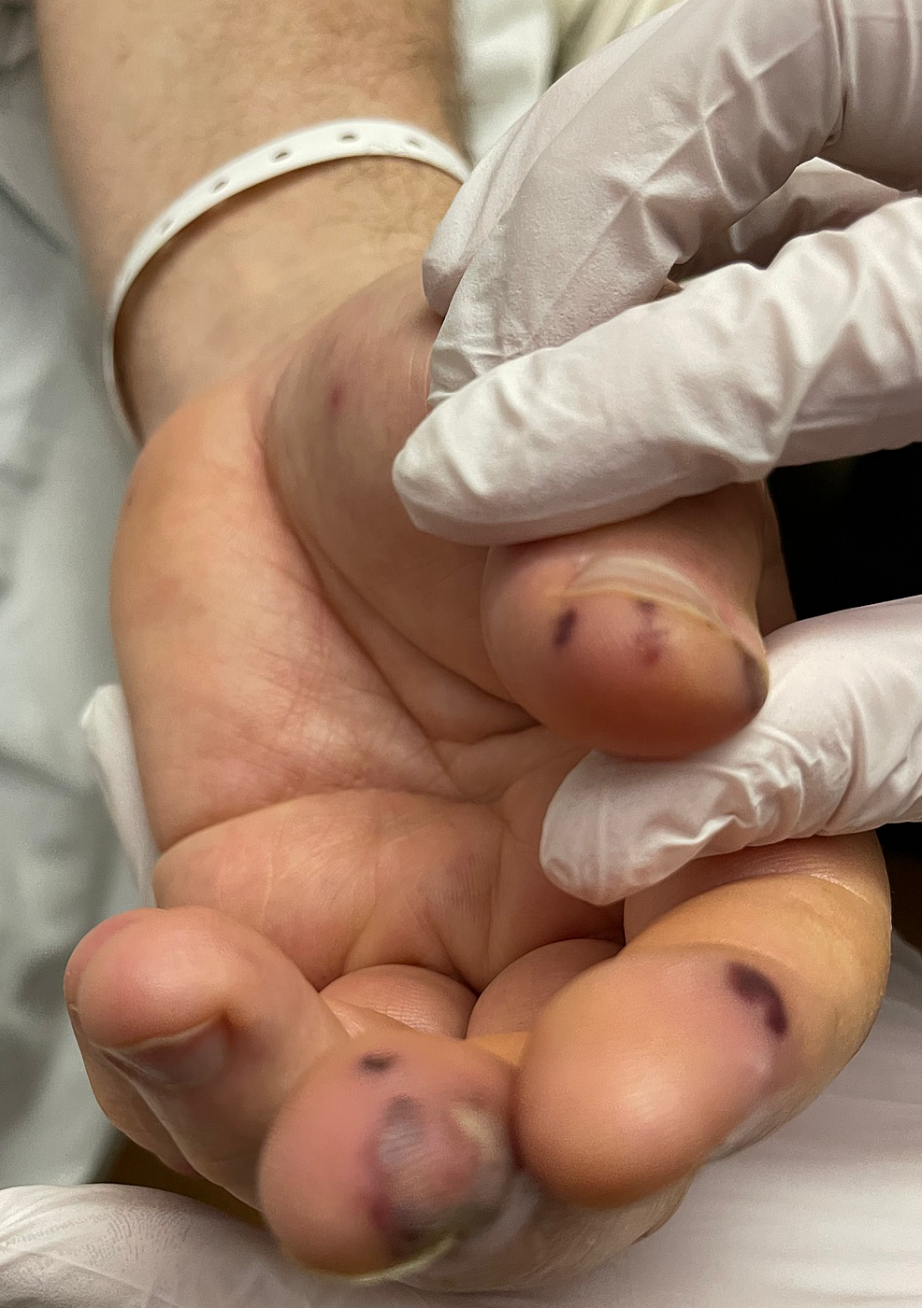
Multiple tender purple lesions, consistent with Osler node in the left hand

**Figure 4:**
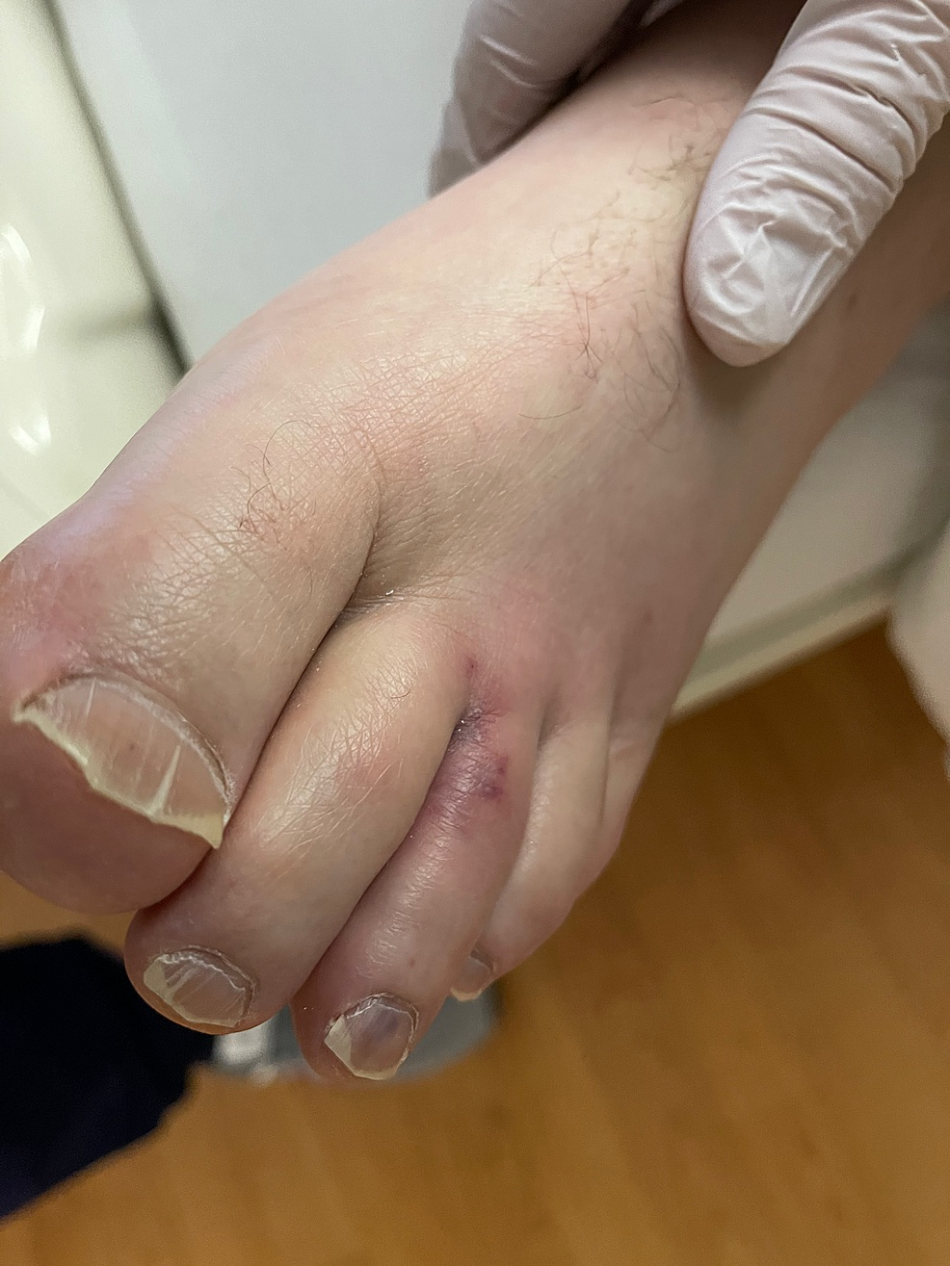
Left foot with splinter hemorrhage and red-purple tender nodule consistent with Osler nodes

On arrival, the patient was afebrile with vitals within normal limits. The patient had a regular rate and rhythm with S1 and S2 present, no murmurs, rubs, or clicks were appreciated. He had multiple erythematous macular lesions in his palms and feet and red-purple nodules that were tender to palpation. He also had splinter hemorrhages in bilateral hand nails and feet (Figures 1-4). The rest of the physical exam was unremarkable.

The patient's initial chest pain was treated for chest wall pain but developed conversational dyspnea, generalized weakness, and fevers at home. Pulmonary embolism was ruled out based on a recent presentation. The patient had multiple risk factors for coronary artery disease and heart failure that could partially explain his symptoms. The presence of fever at home with generalized weakness and physical exam findings also added pericarditis, myocarditis, endocarditis, vasculitis to the differential. 

**Figure 5:**
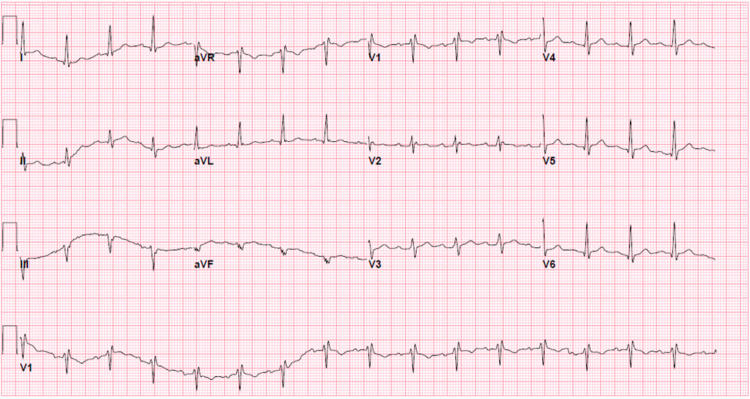
EKG demonstrating normal sinus rhythm with incomplete RBBB RBBB - right bundle branch block

The patient's EKG showed a normal sinus rhythm with an incomplete right bundle branch block without significant ST - T changes (Figure 5). Initial, pertinent labs are summarized in Table 1.

**Table 1:** Summary of initial pertinent labs WBC - white blood cells; CRP - C-reactive protein; ESR - erythrocyte sedimentation rate

Lab (reference value)	Initial value	Follow value up (if applicable)				
Troponin I (<0.028 ng/mL)	0.527	0.735	0.820	0.971	1.284	0.185
WBC (4.00 - 12.00 X 10^3^/mcL)	19					
CRP (<0.50 mg/dL)	25					
ESR (<20 mm/h)	52					
Procalcitonin (≤0.25 ng/ml)	2.88					

**Figure 6:**
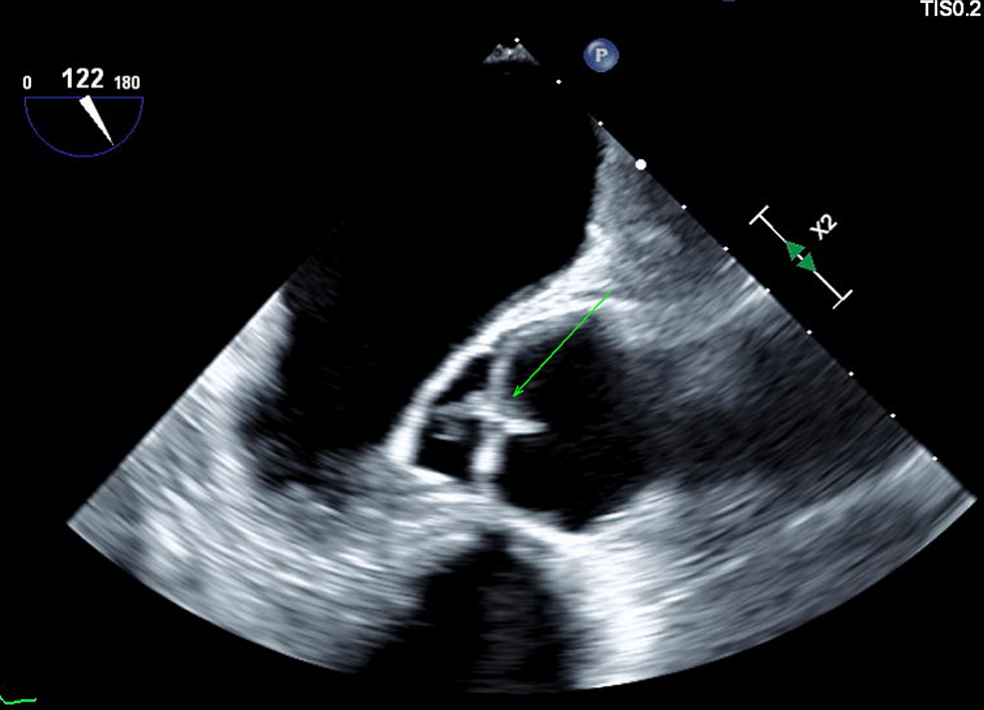
Vegetation noted in non-coronary cusp as a mobile mass with independent motion

The patient had a blood culture and urine culture that both grew methicillin-susceptible *Staphylococcus aureus. *The initial transthoracic echocardiogram (TTE) showed a left ventricular ejection fraction (LVEF) of 60-65% with normal left ventricle (LV) function and no valvular disease. However, the transesophageal echocardiogram (TEE) found a small globular mass with independent motion on the ventricular side of the non-coronary cusp suggestive of vegetation with trace to mild aortic regurgitation (Figure 6).

The patient was diagnosed with aortic valve endocarditis after a careful review of history and physical exam findings, positive blood cultures, and findings of vegetation on an aortic valve on TEE. The patient was found to have left axillary candida intertrigo with superimposed bacterial infection and was likely the source of bacteremia leading to endocarditis. The hospital course was complicated by septic embolic events to the brain with hemorrhagic conversion, pyelonephritis with early abscess formation, T4-T5 discitis with osteomyelitis, and new-onset atrial fibrillation. Surgical intervention was not pursued as the patient would have required high heparin dosing and was at a high risk of further hemorrhagic conversion of septic emboli. The patient was treated with IV cefazolin and then was discharged with IV nafcillin for six weeks. 

**Figure 7:**
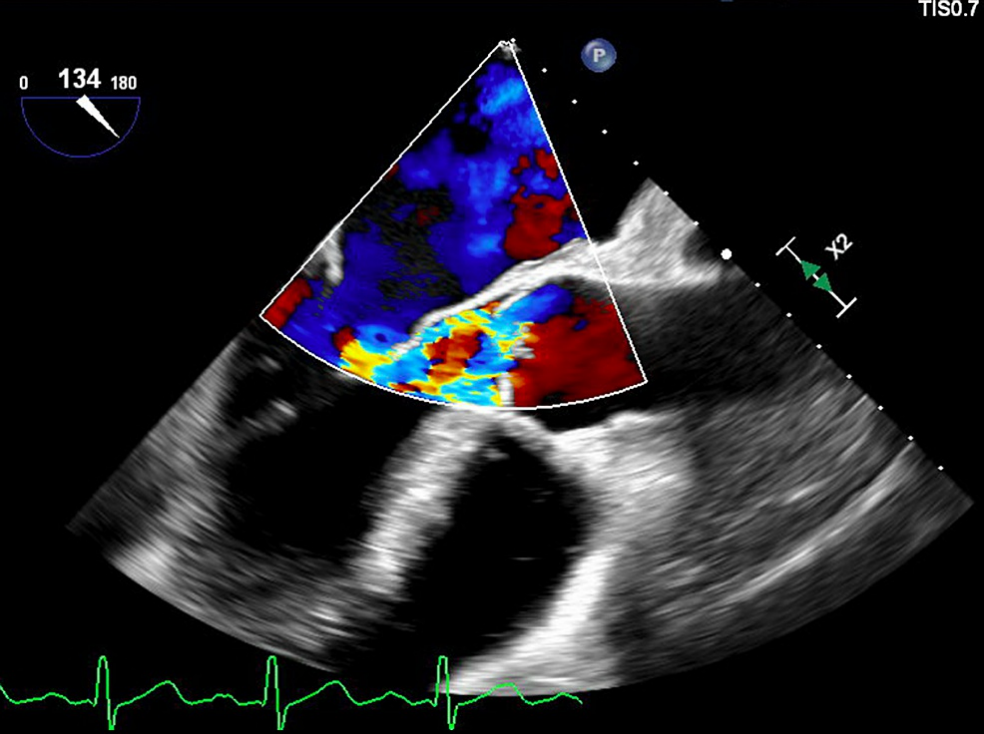
Perforation of the non-coronary cusp with severe aortic valve regurgitation

Unfortunately, the patient presented four days after discharge with worsening dyspnea, hypoxia, and lower extremity swelling. The patient had a repeat TEE that showed vegetations on the right and non-coronary cusp with 0.5 cm and 0.6 cm size, respectively. Perforation of the non-coronary cusp with severe aortic valve regurgitation was also noted (Figure 7). Further neurological workup was pursued, and after weighing the risk and benefits of valve replacement, surgical aortic valve replacement (SAVR) was pursued. The initial post-op course was as expected without significant acute complications. The patient currently has ongoing management for atrial fibrillation and flutter and SAVR with general cardiology, electrophysiology, and cardiothoracic surgery.

## Discussion

Symptoms of endocarditis include fever, chills, anorexia, weight loss, malaise, headache, myalgias, arthralgias, night sweats, abdominal pain, and dyspnea [2]. Risk factors for IE include age over 60 years, male gender, IV drug use, poor oral dentition, and underlying comorbid conditions, including structural heart disease and valvular disease [3]. Physical exam findings include cardiac murmurs, petechiae, splinter hemorrhages, Janeway lesions, Osler nodes, and Roth spots. IE can be diagnosed based on pathology or the modified Duke's criteria, which is based on clinical findings [2]. Positive blood cultures with a particular caveat and echocardiographic evidence of endocardial involvement are the major criteria of the modified Duke's criteria. Minor criteria include predisposition factors, fever, vascular phenomena, immunologic phenomena, and microbiology evidence.

Janeway lesions, Osler's nodes, and Roth spots are unique findings in endocarditis. Janeway lesions are erythematous macular lesions that are non-tender and histologically are microabscesses in the capillaries with neutrophil infiltration [4]. Osler nodes are red-purple or violaceous raised cutaneous nodules that are tender and represent vascular occlusion by microthrombi with immune-mediated vasculitis. Roth spots are exudative, edematous hemorrhagic lesions of the retina with pale centers with a similar underlying mechanism to Osler nodes [4]. An observational, prospective population-based study of 497 patients with definite IE modified Duke criteria with known 487 dermatological status found the prevalence of Osler nodes in 2.7% of the cases, while Janeway lesions were observed in 1.6% of the cases [5]. The study also found that patients with skin manifestations had a higher rate of IE-related extracardiac complications; these were particularly cerebral but overall without increased mortality. Cutaneous palpable purpura lesions have a broad differential, most importantly small to medium vessel vasculitis, drug eruptions including glucocorticoid-induced purpura, arthropod bites, calciphylaxis, and sun exposure [6]. Therefore, establishing the underlying cause of dermatological findings can be difficult and require careful review of the history of present illness, review of systems, and biopsy. Given the low prevalence of Janeway lesions and Osler's nodes in IE cases and broad differential when present, it is not surprising that the sensitivities and specificities of these findings were not available in the literature during our research. 

In our patient case, cardiology service was consulted initially due to concerns for the acute coronary syndrome (ACS) in the setting of chest pain and elevated troponin. However, the patient also met three minor criteria for IE on presentation: fever of 101℉ at home, presence of vascular phenomena with Janeway lesions, and immunological phenomena with splinter hemorrhage and Osler nodes. Clinical experience of the providers involved and suspicion for IE led to further workup, including blood cultures that were positive for methicillin-sensitive Staphylococcus aureus (MSSA) and TEE that found aortic valve vegetation. Therefore, meeting clinical criteria for endocarditis, unfortunately, the patient had other manifestations and complications of IE, including septic emboli to brain and kidney, as well as later rupture of aortic valve cusp. 

Although the presence of vague chest pain is not uncommon in endocarditis, the underlying mechanism is not well described in the literature. It perhaps may simply be due to endocardial inflammation or due to complications such as coexisting pericarditis or septic emboli causing inflammation in the lung parenchyma and pleura. Nevertheless, while it was important to pursue diagnostic workup for coronary artery disease (CAD) during this patient's initial presentation, having a wide differential and early recognition of infective endocarditis may have altered the clinical progression of IE and outcome of this case. Therefore, we share this case to encourage our colleagues to familiarize and recognize the clinical presentation of IE.

## Conclusions

In a patient with chest pain and significant risk factors for coronary artery disease, the acute coronary syndrome is a never-miss diagnosis. However, clinicians should keep their differential broad and consider other diagnoses. In this case, the patient met three minor criteria for infective endocarditis on presentation, which raised suspicion and led to the workup to diagnose infective endocarditis. We hope this case will help and encourage our colleagues around the world to familiarize and recognize IE based upon history and physical examination and lead to early treatment and reduction of disease burden. 
